# Supplementary material for: Patterns of cooperation during collective emergencies in the help-or-escape social dilemma
Source: Sci Rep. 2016 Sep 15;6:33417. doi: 10.1038/srep33417 (PMC5024123; doi:10.1038/srep33417)
Supplement: Supplementary Information [file srep33417-s1.pdf]

Patterns of cooperation during collective emergencies in the help-or-escape social dilemma

Mehdi Moussaïd[1*] and Mareike Trauernicht[1]

[1] Center for Adaptive Rationality, Max Planck Institute for Human Development, 14195, Berlin, Germany.
*Corresponding author: moussaid@mpib-berlin.mpg.de

Supplementary Information

Analysis of the intermediate condition.

In addition to the baseline and the emergency condition, we also conducted an intermediate condition in which participants experienced a moderate level of time and monetary pressure. The parameters for this condition were set to $\alpha = 0$, $\beta = -1$, and $\tau = 30s$.

As for the baseline and the emergency conditions, we also exposed subjects to an introductory framing story for the intermediate condition. The scenario took place in a train station as well. We asked subjects to imagine the following scenario: There is smoke in the train station because a fire seemed to have broken out. To not suffer any damage, they need to leave the building soon. Similar to the other condition, they meet people who need their help on their way out. Helping these individuals decreases their own chances to escape without harm, but increases the other individuals' chances to escape. To point out moderate stress, we told participants there is only smoke but no fire and they are unsure about the best way out of the building.

In the intermediate condition, subjects helped on average 3.68 (SD = 2.19) people, and gave on average 44.7% (*SD* = 25.04) of their chance of success to help others. The absolute and effective processing times are shown in the **figure S1**.

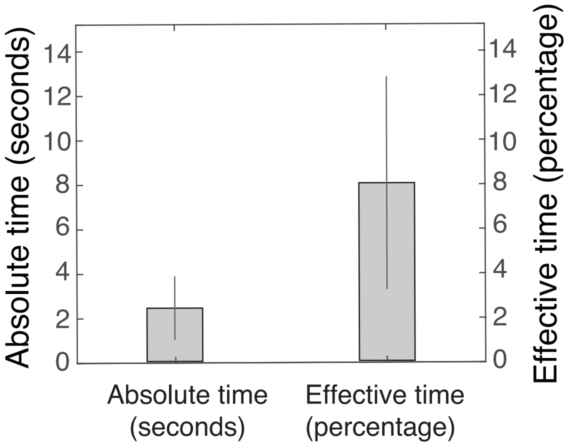

Figure S1: **Processing time**. Average time spent in the game setting without helping anybody in the intermediate condition, for all 104 participants. The absolute time is measured in seconds, whereas the effective time is measured in terms of lost chances to escape. Errors bars indicate the standard deviation of the mean.

Overall, these results are consistent with those presented in the main text. First, participants in the intermediate condition tend to give about the same amount of chances to help others as in the two other conditions. A one-way ANOVA comparing participants' cooperation at the process level yielded no significant effect of the experimental conditions [$F(2, 309) = 0.19$, $p = 0.825$]. This confirms the use of an egalitarian heuristic, whereby participants would help others until their own success chance aligns with the one of the group (i.e. 60%). Participants in the intermediate condition spent on average 2.4 seconds (SD = 1.4) for motor actions and thinking delays, which is equivalent to a loss of 8.0% (SD = 4.8) of their success chance (given the decay rate of approximately 3.5% per second). Therefore, among the average 44.7% of chance that participants are willing to give to help others, only 36.7% (corresponding to 44.7 – 8) can be effectively used. The cost of helping being 10% of the helper's chances, participants in this condition could help 3.67 persons on average, which explains the observed average of 3.68 helped persons in this condition.
Therefore, the mechanisms described in the main text are also consistent with the results of the intermediate condition.

Study of potential habituation effects.

One possible drawback of the fixed condition order that we choose to implement is the possible interaction with habituation effect. In fact, participants in our experiment are necessarily exposed to the emergency condition at the end of the experiment. Therefore, tiredness or habituation to the design could be confounded with the experimental condition. To address this issue, we looked at the temporal sequence of rounds, in the order in which they were presented to the participants (**Figure S2**). At the process level, participants consistently took the same risk to help others round after round, and across conditions. No

progressive change can be observed, as one would have expected in case of habituation effects. Formally, a one-way ANOVA comparing the process level cooperation between rounds, irrespective of the condition indicated the absence of significant changes [$F(14, 1545) = .27$; $p = .99$]. The stability of the helping behaviors is in line with our main findings: participants took the same risk to help (approximately 40%) in all conditions. This finding is thus devoid of habituation effects.

At the outcome level, the same consistency is visible across the baseline and the intermediate condition, but is followed by a qualitative shift at the beginning of the emergency condition. A one-way ANOVA indicated no significant changes across the first 10 rounds (i.e. for the baseline and the intermediate condition [$F(9, 1030) = .05$; $p = 1$]), and no changes across the last 5 rounds (i.e. for the emergency condition [$F(4, 515) = .17$; $p = .95$])). Clearly, this sudden shift is not due to habituation effects (which would have rather produced a gradual change over rounds), but results from the specificities of the emergency condition. Again, this shift is in line with our findings suggesting that the time pressure of the emergency conditions did not allow participants to help as many people as in the two preceding conditions, despite taking the same risk for helping others. Habituation effects, therefore, are unlikely to have affected our findings.

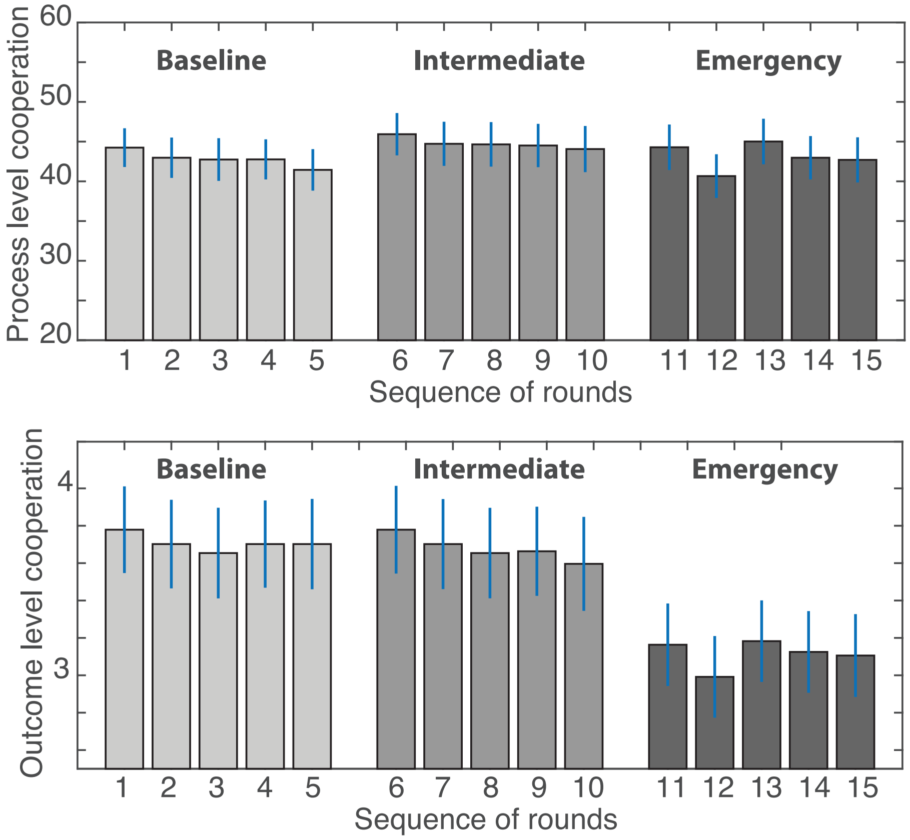

Figure S2: Control for habituation effects. (Top) The average risk taken to help others (process level) in each round, for all 104 participants. The values along the y-axis indicate

the percentage of success chance that participants gave to help others. No progressive change is visible over rounds, suggesting the absence of habituation effects. (Bottom) The average number of person helped (outcome level) in each round, for all 104 participants. The sudden shift occurring at the beginning of the emergency condition is caused by the strong time pressure associated to that condition. Again, no gradual change is visible across rounds. Blue bars indicate the standard error of the mean. Rounds are numbered according to the order in which they were presented to the participants.
